# Knowledge of Vaginal Microbiota and Its Association with Perceptions of Vulvovaginal Aesthetic Procedures Among Saudi Women

**DOI:** 10.3390/healthcare13161955

**Published:** 2025-08-09

**Authors:** Esraa Aldawood, Lama Alzamil, Layla Faqih, Sarah Almuhayya

**Affiliations:** Department of Clinical Laboratories Sciences, The College of Applied Medical Sciences, King Saud University, Riyadh 12372, Saudi Arabia; lalzamil@ksu.edu.sa (L.A.); lfaqih@ksu.edu.sa (L.F.); salmohayya@ksu.edu.sa (S.A.)

**Keywords:** vaginal microbiota, microbiome knowledge, vulvovaginal esthetic procedures, women health

## Abstract

Background: The human microbiome includes trillions of microorganisms, with the vaginal microbiota playing a vital role in women’s reproductive health. Concurrently, interest in vulvovaginal esthetic procedures (VVEP) is increasing. This study aimed to compare single and married or previously married women in terms of their knowledge of the human microbiome, particularly the vaginal microbiota, and their perceptions of VVEP. It also examined associations between microbiota awareness, attitudes toward VVEP, and sociodemographic factors. Methods: A cross-sectional, online survey was distributed to women aged 18 years and older in Saudi Arabia. A total of 1019 responses were collected. Chi-square tests compared knowledge responses between marital groups, while linear regression was used to explore associations between microbiota knowledge and participant characteristics. Results: Single women showed greater awareness of general microbiome concepts, with 42% correctly identifying the term “microbiome” compared to 29.89% of married or previously married women. In contrast, married or previously married women demonstrated better knowledge of vaginal microbiota and the effects of antibiotic misuse. Of the participants, 6.6% had undergone one or more VVEP. Furthermore, 19.7% of single women and 18.3% of married or previously married women expressed future interest in undergoing such procedures. Marital status influenced perceptions of specific procedures, with married or previously married women more likely to justify interventions such as augmentation of the labia minora/“G-spot” augmentation and vaginal rejuvenation. Justification for augmentation of the labia minora/“G-spot” augmentation in this group was associated with lower vaginal microbiota knowledge. Healthcare professionals exhibited significantly higher microbiota awareness. Conclusion: Enhancing women’s knowledge of vaginal microbiota can enhance informed decision-making and reduce unnecessary esthetic interventions among Saudi women, thereby supporting better reproductive health outcomes.

## 1. Introduction

The human microbiome comprises trillions of microorganisms, including bacteria, viruses, and fungi, that inhabit various sites of the human body, playing crucial roles in maintaining health, modulating immunity, and influencing disease processes [1]. Among these diverse microbial communities, the vaginal microbiota holds particular significance for women’s reproductive health.

In approximately 70% of women of reproductive age, the vaginal microbiota is dominated by *Lactobacillus* species, which help maintain vaginal health by producing lactic acid [2]. This creates an acidic environment that inhibits pathogenic growth, thereby preventing infections and preserving the overall stability of the vaginal ecosystem [3]. Disruption of this microbial balance due to factors such as menstruation, sexual activity, pregnancy, or infection can increase microbial diversity, and lead to conditions such as bacterial vaginosis, which is associated with adverse outcomes such as preterm birth or infertility [4,5,6,7,8,9]. In addition to the key role of *Lactobacillus*-dominated communities in protecting the vagina against pathogen colonization, they also play a role in shaping the neonatal microbiome during childbirth, supporting the development of a healthy gut and skin microbiota in the newborn [10].

Despite its clinical significance, studies concerning public knowledge of the composition, function, and maintenance of the vaginal microbiome are lacking, particularly in Saudi Arabia. Most local studies that have examined general microbiome knowledge have revealed suboptimal awareness of microbiota-related topics across various population groups [11,12]. For instance, among dental professionals, while 94.6% had heard of the term “microbiome,” only 6.7% understood its systemic implications [11]. Similarly, medical sciences students in Saudi Arabia showed low to moderate understanding of microbiome dysbiosis and its role in allergic diseases [12]. Furthermore, only 11.8% of adolescent girls in Riyadh demonstrated good knowledge about abnormal vaginal discharge, with many resorting to self-treatment or avoiding care due to fear or embarrassment [13]. Concurrently, there has been growing interest in vulvovaginal esthetic procedures (VVEP), including vaginal rejuvenation, labiaplasty, vulvar liposculpturing, and G-spot amplification. These trends reflect evolving societal norms, increased body image awareness, and shifting perceptions of femininity. However, the credibility and medical justification for such procedures remain widely questioned within the medical community [14]. In 2006, the American College of Obstetricians and Gynecologists (ACOG) issued a statement indicating that vaginal rejuvenation and other female genital cosmetic surgeries are not considered standard surgical practices due to a lack of evidence regarding their safety and efficacy, in addition to potential complications such as infection, dyspareunia, scarring, and altered sensation. Similar positions have been adopted by the Royal Australian and New Zealand College of Obstetricians and Gynaecologists and the Society of Obstetricians and Gynaecologists of Canada [15]. A local study targeted health professionals and medical students who showed inconsistent understanding and ethical consideration of VVEP, with many calling for more education and inclusion of these topics in medical curricula [16]. While these procedures are becoming increasingly popular, women’s motivations, expectations, and personal experiences remain inadequately studied. In Saudi Arabia, interest in such procedures is shaped by a complex interplay of cultural, social, and personal factors, yet comprehensive data on women’s attitudes and experiences are still lacking.

This study seeks to bridge these gaps by comparing single and married or previously married women, first by assessing their knowledge of the human microbiome, with a specific focus on vaginal microbiota. It will also explore women’s perceptions, motivations, and experiences regarding VVEP, providing insight into the personal, social, and cultural factors shaping these choices. Additionally, the study aims to examine potential correlations between women’s awareness of vaginal microbiota and their attitudes toward VVEP, hypothesizing that greater vaginal microbiota knowledge may influence perceptions and acceptance of such interventions. Recognizing that knowledge and attitudes may vary across population groups, the research will further assess the impact of sociodemographic factors on women’s understanding of microbiota. The findings are expected to inform targeted educational initiatives, enhance patient counseling, and support public health strategies aimed at promoting reproductive health literacy and evidence-based, informed decision-making among women.

## 2. Materials and Methods

### 2.1. Study Design and Setting

A cross-sectional online survey-based study was conducted by distributing a survey link to women aged 18 years and older residing in Saudi Arabia. The required sample size for this study was calculated to be 377 participants using Raosoft, Inc. (Seattle, WA, USA) (http://www.raosoft.com/samplesize.html) (accessed on 26 June 2024), with a 95% confidence level and a 5% margin of error. However, data collection continued until 1019 women had responded.

### 2.2. Data Collection

The study was conducted from December 2024 to January 2025 using a self-administered, structured questionnaire disseminated via an online link. A pilot study involving 20 women was conducted to assess the item clarity, comprehension, and the estimated time required to complete the survey Based on the pilot results, no major changes were needed. The questionnaire was developed in English, translated into Arabic, and then back-translated by a bilingual expert to ensure linguistic and conceptual equivalence.

The response format for items assessing knowledge of the human microbiome and vaginal microbiota was True, False, or “I Don’t Know”. The scoring method was informed by tools previously validated in HPV knowledge surveys [17,18]. Responses of “I Don’t Know” were considered incorrect and assigned a score of 0. Correct answers received a score of 1, and the total knowledge scores were calculated as the sum of correct responses. Higher scores indicated greater knowledge.

The questionnaire consisted of four sections:

1. Sociodemographic Information: This included questions on age, marital status, education level, occupation, employment in the healthcare sector, and region of residence in Saudi Arabia.
2. Human Microbiome Knowledge: This section included eight items designed to evaluate general knowledge of the human microbiome. Sample statements included “The term ‘Microbiome’ refers to all microorganisms in the human body,” “Microbiome composition is similar for all people,” “There are microorganisms living naturally in the intestinal tract,” “There are microorganisms living naturally in the respiratory tract,” “There are microorganisms living naturally on the skin,” “There are microorganisms living naturally in the vagina,” “All microorganisms found on the human body are harmful,” and “Antibiotic misuse can negatively impact the microbiota.” Items were partially adapted from validated instruments used in previous microbiome knowledge studies [12,19,20]. For example, the item “All microorganisms found on the human body are harmful” was validated in [19], and the item “Antibiotic misuse can negatively impact the microbiota” was validated in [20]; the remaining items were adapted from an instrument applied in a previous microbiome awareness survey [12]. In this study, the internal consistency reliability of the human microbiome knowledge section, as measured by Cronbach’s alpha, was 0.72, indicating acceptable reliability.
3. Vaginal Microbiota Knowledge: This section comprised five items developed specifically for this study to assess the understanding of the vaginal microbiota. The items were informed by the current literature and the expertise of microbiome researchers. Content validity was confirmed by a panel of three academic microbiologists, who reviewed the items for clarity, scientific accuracy, and relevance; no revisions were necessary following their evaluation. Statements included “The vaginal microbiome plays a crucial role in maintaining vaginal health and defending against pathogenic microorganisms,” “The vaginal microbiome is dynamic and dominated by *Lactobacillus* in healthy individuals,” “The composition of the vaginal microbiota can change throughout a woman’s menstrual cycle,” “Use of antibiotics can cause temporary changes in the vaginal microbiota,” and “Douching can disrupt the vaginal microbiota by altering the microbial community.” Internal consistency reliability for this scale was acceptable, with a Cronbach’s alpha of 0.73.
4. Perceptions of VVEP: This section included six items adapted from a validated study [16] to assess participants’ perceptions of VVEP and whether they had undergone or were considering undergoing any of these procedures. The procedures assessed were augmentation of the labia minora/‘G-spot’ augmentation, clitoral surgery, vaginal rejuvenation, augmentation of the labia majora, mons pubis liposuction, laser vaginal tightening/whitening, and vaginal laser treatment for atrophy. Participants rated the justification for each procedure using a four-point scale: Highly justified, Justified, Rarely justified, and Not justified.”

After obtaining ethical approval, the survey link was created using Google Forms and distributed to participants. The inclusion criteria were women aged 18 years or older residing in Saudi Arabia, while males and individuals under 18 years of age were excluded. Participants were recruited using convenience sampling. Data collectors from various regions of Saudi Arabia assisted in distributing the survey link, primarily via WhatsApp groups. To ensure data completeness, all questions in the online survey were mandatory; participants could not proceed or submit the form without answering every item. As a result, the final dataset comprised 1019 fully completed responses included in the analysis.

### 2.3. Ethical Consideration

Participants were informed about the study’s objectives and provided voluntary consent before completing the questionnaire. Responses were collected anonymously and kept confidential. The study received ethical approval from the Institutional Review Board at King Saud University (Ref. No. 24/1508/IRB) (approval date: 22 August 2024). All data were accessible only to the research team.

### 2.4. Statistical Analysis

Percentages were calculated for categorical variables. The Chi-square test was used to compare the answers of knowledge responses between single and married or previously married women and to assess differences in their justifications for VVEP. To analyze the association between knowledge of the vaginal microbiota/human microbiome and participant characteristics, linear regression analysis was conducted, using the total knowledge score as the dependent variable and justification of VVEP/sociodemographic factors as independent variables. Adjusted beta estimates and 95% confidence intervals (CIs) were reported. Data analysis was performed using GraphPad Prism 9.

## 3. Results

### 3.1. Sociodemographic Characteristics of the Respondents

All 1019 submitted responses were complete and included in the final analysis. As shown in Table 1, the majority of respondents (33.76%) were between 20 and 30 years old, followed by those under 20 years (24.14%), while only 7.36% were over 51 years old. Regarding marital status, more than half of the respondents (54.37%) were single, while 45.63% were either married or had been previously married. In terms of educational background, most participants (65.26%) held a bachelor’s degree, followed by high school graduates (22.37%). A smaller proportion had postgraduate degrees (10.21%), while 2.16% had only an elementary-level education. Concerning occupational status, the majority were students (43.18%), followed by full-time employees (23.55%), and unemployed individuals (21.39%). Other categories included self-employed individuals (3.93%), part-time employees (2.94%), and retired individuals (5.00%). A notable 22.47% of respondents worked in the healthcare sector, whereas the majority (77.53%) were from non-healthcare backgrounds. Regarding geographical distribution in Saudi Arabia, most participants lived in the central region (69.09%), while 10.50% were from the eastern region, 10.30% were from the western region, 5.79% were from the southern region, and 4.32% were from the northern region.

### 3.2. Participants’ Knowledge of the Human Microbiome and Vaginal Microbiota

The participants demonstrated varying levels of knowledge regarding the human microbiome, as shown in Table 2. While more than 80% of both single and married or previously married women correctly identified that not all microorganisms found on the human body are harmful and that microorganisms naturally inhabit the intestinal tract, nearly 47% of participants from both groups failed to recognize the presence of microorganisms in the respiratory tract.

Notable differences in knowledge between single and married or previously married women were observed (Table 2). A significantly higher proportion of single women (42.06%) correctly identified that the term microbiome refers to all microorganisms in the human body, compared to 29.89% of married or previously married women (*p* < 0.0001). Similarly, significantly more single participants (46.57%) recognized that microbiome composition varies among individuals, compared to married or previously married women (39.35%, *p* = 0.0206). Additionally, a significantly greater proportion of single women (77.44%) correctly acknowledged that microorganisms naturally exist on the skin, compared to 67.53% of married or previously married women (*p* = 0.0004). Conversely, knowledge about the vaginal microbiota was significantly higher among married or previously married women, with 78.28% correctly identifying the presence of microorganisms in the vagina, compared to 66.61% of single women (*p* < 0.0001). Furthermore, awareness of the negative impact of antibiotic misuse on microbiota was significantly greater among married or previously married women (76.13% vs. 66.97%, *p* = 0.0013).

Regarding knowledge of vaginal microbiota, more than half of the participants understood that the vaginal microbiome plays a crucial role in maintaining vaginal health. However, knowledge of its specific characteristics was limited. Only 16.25% of single women and 14.84% of married or previously married women correctly identified that a healthy vaginal microbiome is dominated by *Lactobacillus.* Similarly, awareness that the vaginal microbiota composition fluctuates throughout the menstrual cycle was low, with 43.86% of single and 43.87% of married women answering correctly.

Nevertheless, married or previously married women demonstrated significantly greater knowledge of the effects of antibiotic use on vaginal microbiota (65.38% vs. 54.15%, *p* = 0.0003). Additionally, they exhibited significantly higher awareness of the impact of douching on vaginal microbiota (60.65% vs. 51.26%, *p* = 0.0027).

### 3.3. Perceptions of Female Genital Cosmetic Surgery Among Participants

Participants were asked about their opinions regarding esthetic procedures related to vulvovaginal modifications (Table 3). Overall, the majority of procedures were perceived as “not justified” by both groups. However, augmentation of the labia minora/“G-spot” augmentation was more frequently considered as justified by married or previously married women than by single participants (11.40% vs. 6.50%, *p* = 0.0267). Similarly, vaginal rejuvenation was significantly more accepted among married or previously married women, with 12.26% considering it highly justified compared to 6.32% of single participants (*p* < 0.0001). In contrast, perceptions regarding clitoral surgery, labia majora augmentation, mons pubis liposuction, laser vaginal tightening/whitening, and vaginal laser for atrophy did not differ significantly between the two groups (*p* > 0.05).

**Figure 1:**
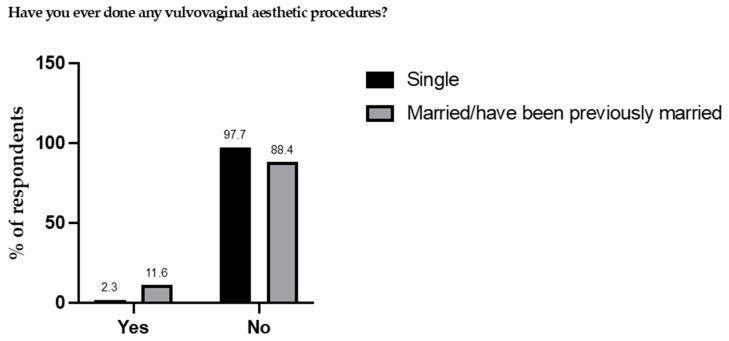
Percentages of women who have undergone vulvovaginal esthetic procedures.

When participants were asked if they had ever undergone any VVEP (Figure 1), the vast majority answered no (97.7% of single women and 88.4% of married or previously married participants). Correspondingly, 2.3% of single women and 11.6% of married or previously married women reported having undergone one or more VVEP. Based on these subgroup percentages and sample sizes (554 single and 465 married or previously married women), the overall weighted prevalence of women who had undergone VVEP in our sample was calculated as 6.6%.

When asked whether they would personally consider undergoing these procedures, most respondents in both groups stated they would not (80.32% of single participants and 81.72% of married or previously married participants) (Table 4). However, married or previously married women showed a higher willingness to undergo vaginal rejuvenation (7.96% vs. 1.99%) and laser vaginal tightening (5.38% vs. 2.71%) compared to single women. Conversely, single women exhibited a greater inclination toward whitening procedures (15.16% vs. 9.68%) (Table 4).

### 3.4. Association Between Vaginal Microbiota Knowledge and Perception of Vulvovaginal Esthetic Procedures

The association between participants’ knowledge of vaginal microbiota and their justification for vulvovaginal esthetic procedures, stratified by marital status, was assessed (Table 5). Among married or previously married women, those who considered augmentation of the labia minora/G-spot augmentation as “justified” showed a significantly lower vaginal microbiota knowledge score (β = −0.6958, 95% CI: −1.369 to −0.02231, *p* < 0.05). Conversely, those who perceived clitoral surgery as “justified” had significantly higher knowledge scores (β = 0.6982, 95% CI: 0.1151 to 1.281, *p* < 0.05). Additionally, augmentation of the labia majora was positively associated with significantly higher microbiota knowledge scores among married or previously married women who considered the procedure to be “highly justified” (β = 1.216, 95% CI: 0.01080 to 2.422, *p* < 0.05).

Regarding vaginal rejuvenation, single women who considered the procedure as “highly justified” (β = 1.054, 95% CI: 0.3493 to 1.759, *p* < 0.01), “justified” (β = 0.5809, 95% CI: 0.1764 to 0.9854, *p* < 0.01), or “rarely justified” (β = 0.5851, 95% CI: 0.2030 to 0.9673, *p* < 0.01) demonstrated significantly higher vaginal microbiota knowledge compared to those who did not consider the procedure justified. However, no significant association was observed for married or previously married women. No significant associations were observed for mons pubis liposuction, laser vaginal tightening/whitening, or vaginal laser treatment for atrophy across both marital groups.

### 3.5. Association Between General Microbiota Knowledge and Sociodemographic Characteristics

The associations between general microbiota knowledge and various sociodemographic characteristics were examined (Table 6). Age, marital status, education, occupation, and geographic region did not show significant associations with microbiota knowledge. However, participants who were working in the healthcare sector exhibited significantly higher microbiota knowledge scores (β = 1.213, 95% CI: 0.8923 to 1.533, *p* < 0.0001) compared to non-healthcare professionals.

## 4. Discussion

Given the importance of educating women about the vaginal microbiota, this study addresses a critical gap in women’s health by assessing the awareness and understanding of the human microbiome, with a particular focus on vaginal microbiota. In light of the growing popularity of VVEP, we explored the association between women’s vaginal microbiota knowledge and their perceptions of these procedures among both single and married or previously married women. Additionally, we examined how sociodemographic characteristics influence microbiome knowledge to identify potential determinants of awareness in this area.

Regarding human microbiome knowledge, more than 80% of both single and married or previously married participants recognized that not all microorganisms on the human body are harmful and that microorganisms naturally inhabit the intestinal tract. This suggests a higher level of awareness compared to Singaporean adults, where only 32.6% had ever heard of the gut microbiota [21]. However, nearly half of the participants in our study failed to recognize the presence of microorganisms in the respiratory tract, indicating a partial understanding of the microbiome, likely shaped by the predominant public discourse on gut health [22,23].

Differences in microbiome knowledge were observed based on marital status. Single women demonstrated greater awareness of general microbiome concepts compared to their married or previously married counterparts. Specifically, 42% of single women correctly identified the term “microbiome,” compared to 29.89% of married or previously married women. Both groups; however, showed higher recognition than participants in a U.S. study, where only 21.8% recognized the term [24]. Single women were also more likely to understand inter-individual variability in microbiome composition and the presence of microorganisms on the skin. This may be attributed to the fact that many single women were students who are potentially more exposed to scientific content through academic settings or social media. These findings align with a study conducted in Jordan, which reported higher microbiota knowledge among university students [25]. Conversely, married or previously married women exhibited greater knowledge about the vaginal microbiota and the negative effects of antibiotic misuse. This could be attributed to their more frequent gynecological healthcare visits, which may expose them to more information related to vaginal health. Although most participants in the present study were from the Central region, related studies conducted in Makkah [26] and in Riyadh [13] similarly identified limited awareness and inadequate self-care practices concerning abnormal vaginal discharge. These findings underscore persistent gaps in vaginal health literacy across different regions of Saudi Arabia.

Female genital cosmetic surgery is defined as a set of non-medically indicated surgical procedures that alter the structure and appearance of healthy external genitalia [27]. While most participants in this study perceived these procedures as unjustified, 6.6% had already undergone one or more such procedures, and approximately 20% from each group considered undergoing them in the future. This prevalence is comparable to that reported by Alrashed et al. (2023) conducted in Saudi Arabia, where 7.6% of women had undergone female genital cosmetic surgery, and 19% expressed consideration of such procedures [28]. Our findings indicate that marital status influenced perceptions of specific interventions such as augmentation of the labia minora/“G-spot” augmentation and vaginal rejuvenation, with married or previously married women being more likely to justify and consider these procedures. This aligns with national findings showing that Saudi women’s attitudes toward cosmetic surgery are shaped by interpersonal and social influences, including marriage and body image concerns [29]. Interestingly, justification of augmentation of the labia minora/“G-spot” augmentation among married or previously married women was associated with lower vaginal microbiota knowledge. This inverse relationship may reflect a gap between cosmetic motivations and the biomedical understanding of vaginal health. Furthermore, participants working in the healthcare sector demonstrated higher microbiota knowledge, consistent with findings from a UAE study, which identified healthcare professionals as having significantly greater microbiota knowledge than the general population [19]. This highlights the role of education as a protective factor, empowering women to make informed decisions about their reproductive and esthetic health.

The findings of this study hold important implications for both clinical practice and public health education in Saudi Arabia. The observed association between higher microbiota knowledge scores and prior marital status may reflect greater exposure to reproductive healthcare services, such as gynecological consultations or childbirth-related care. These encounters represent critical touchpoints for health professionals to deliver education on vaginal health, emphasizing the importance of the microbiota in maintaining urogenital health and preventing infections. Furthermore, in the Saudi cultural context, where discussions around intimate health remain sensitive, social norms of modesty and stigma may contribute to knowledge gaps and shape attitudes toward VVEP. Some women may perceive these procedures as a way to align with societal ideals of cleanliness, youthfulness, or marital fulfillment, even in the absence of clear medical indications. This highlights the importance of culturally sensitive, evidence-based education to empower women in making informed choices. Additionally, the findings point to an urgent need for regulatory and ethical frameworks to guide the provision of VVEP, ensuring that procedures are not marketed or performed based on unfounded claims or societal pressure. Integrating vaginal microbiota education into routine clinical practice and public health campaigns could play a vital role in enhancing reproductive health literacy and reducing misinformation.

This study has several limitations. Firstly, due to its cross-sectional design, causal relationships between participants’ knowledge and their perceptions cannot be established. Secondly, the use of self-reported data may introduce recall or social desirability bias. Additionally, the convenience sampling strategy and social media-based recruitment may limit the generalizability of the findings. Notably, the sample was markedly skewed toward respondents from the central region of Saudi Arabia (69.09%), which limits the extent to which the results can be generalized to women in other regions. However, the large sample size and variation in participants’ age groups enhance the reliability of observed trends and associations. Another limitation arises from the questionnaire design, which combined certain VVEP such as “augmentation of the labia minora” with “G-spot augmentation” as single response items. This grouping may have obscured nuanced differences in perceptions and experiences related to individual procedures. Future studies should aim to separate these procedures in the survey design to allow for more detailed analysis and a clearer interpretation of women’s attitudes toward specific interventions, and should also aim for regionally balanced sampling to improve the representativeness and generalizability of the findings. To our knowledge, this is the first study in Saudi Arabia to explore women’s knowledge of the vaginal microbiota and the first globally to examine its association with VVEP perceptions.

## 5. Conclusions

These findings highlight the need for targeted educational initiatives that consider sociodemographic differences to enhance microbiota knowledge across diverse segments of the Saudi population. While awareness of VVEP is increasing, comprehensive education addressing both their esthetic and health-related implications remains essential. Improving women’s understanding of vaginal microbiota may positively influence their perceptions and decisions regarding VVEP, promoting informed choices and contributing to better reproductive health outcomes. We recommend implementing culturally sensitive health education campaigns through schools, universities, primary healthcare centers, and digital platforms to raise awareness about the role of the vaginal microbiota and the implications of esthetic procedures. These efforts should also involve healthcare providers to ensure consistent, evidence-based counseling practices.

## Figures and Tables

**Table 1:** Demographic characteristics of the participants (*n* = 1019).

Item	N	%
Age		
18–20 years	246	24.14
20–30 years	344	33.76
30–40 years	187	18.35
40–50 years	167	16.39
>51 years	75	7.36
Marital Status		
Single	554	54.37
Married or have been previously married	465	45.63
Education		
Elementary	22	2.16
High school	228	22.37
Bachelor	665	65.26
Postgraduate	104	10.21
Occupation		
Student	440	43.18
Employed full-time	240	23.55
Employed part-time	30	2.94
Self employed	40	3.93
Unemployed	218	21.39
Retired	51	5.00
In healthcare		
Yes	229	22.47
No	790	77.53
Region		
Central	704	69.09
Western	105	10.30
Eastern	107	10.50
Northern	44	4.32
Southern	59	5.79

**Table 2:** Knowledge of the human microbiome and vaginal microbiota.

Marital Status	Single (*n* = 554)		Married or Have Been Previously Married (*n* = 465)		*p* Value
Statement	Correct	Incorrect	Correct	Incorrect	
Knowledge of the Human Microbiome	N (%)	N (%)	N (%)	N (%)	
1-The term ‘Microbiome’ refers to all microorganisms in the human body.	233 (42.06)	321 (57.94)	139 (29.89)	326 (70.11)	<0.0001
2-All microorganisms found on the human body are harmful.	486 (87.73)	68 (12.27)	394 (84.73)	71 (15.27)	0.1654
3-Microbiome composition is similar for all people.	258 (46.57)	296 (53.43)	183 (39.35)	282 (60.65)	0.0206
4-There are microorganisms living naturally in the intestinal tract.	476 (85.92)	78 (14.08)	388 (83.44)	77 (16.56)	0.2723
5-There are microorganisms living naturally in the respiratory tract.	290 (52.35)	264 (47.65)	248 (53.33)	217 (46.67)	0.7533
6-There are microorganisms living naturally on the skin.	429 (77.44)	125 (22.56)	314 (67.53)	151 (32.47)	0.0004
7-There are microorganisms living naturally in the vagina.	369 (66.61)	185 (33.39)	364 (78.28)	101 (21.72)	<0.0001
8-Antibiotics misuse can negatively impact the microbiota.	371 (66.97)	183 (33.03)	354 (76.13)	111 (23.87)	0.0013
**Knowledge of Vaginal Microbiota**					
1-The vaginal microbiome plays a crucial role in maintaining vaginal health and defending against pathogenic microorganism.	317 (57.22)	237 (42.78)	267 (57.42)	189 (40.65)	0.6696
2-Vaginal microbiome is dynamic, dominated by Lactobacillus in health.	90 (16.25)	454 (83.75)	69 (14.84)	396 (85.16)	0.4586
3-The composition of the vaginal microbiota can change throughout a woman’s menstrual cycle.	243 (43.86)	311 (56.14)	204 (43.87)	261 (56.13)	0.9979
4-Usage of antibiotics can cause a temporal change in human vaginal microbiota.	300 (54.15)	254 (45.85)	304 (65.38)	161 (34.62)	0.0003
5-Douching can disrupt vaginal microbiota by altering microbial community.	284 (51.26)	270 (48.74)	282 (60.65)	183 (39.35)	0.0027

Note: Incorrect and “I Don’t Know” responses were combined and coded as incorrect answers in the analysis.

**Table 3:** Perceptions of vulvovaginal esthetic procedures.

Statement	Single (*n* = 554)	Married or Have Been Previously Married (*n* = 465)	*p* Value
	N (%)	N (%)	
1-Augmentation of the labia minora/“G-spot” augmentation ^a^			0.0267
Highly justified	16 (2.89)	8 (1.72)	
Justified	36 (6.50)	53 (11.40)	
Rarely justified	131 (23.65)	98 (21.08)	
Not justified	371 (66.97)	306 (65.81)	
2-Clitoral surgery			0.8715
Highly justified	13 (2.35)	12 (2.58)	
Justified	83 (14.98)	63 (13.55)	
Rarely justified	166 (29.96)	135 (29.03)	
Not justified	292 (52.71)	255 (54.84)	
3-Vaginal rejuvenation			<0.0001
Highly justified	35 (6.32)	57 (12.26)	
Justified	169 (30.51)	183 (39.35)	
Rarely justified	174 (31.41)	114 (24.52)	
Not justified	176 (31.77)	111 (23.87)	
4-Augmentation of the labia majora			0.4292
Highly justified	15 (2.71)	12 (2.58)	
Justified	45 (8.12)	52 (11.18)	
Rarely justified	103 (18.59)	85 (18.28)	
Not justified	391 (70.58)	316 (67.96)	
5-Mons pubis liposuction			0.1967
Highly justified	9 (1.62)	11 (2.37)	
Justified	77 (13.90)	48 (10.32)	
Rarely justified	149 (26.90)	116 (24.95)	
Not justified	319 (57.58)	290 (62.37)	
6-Laser Vaginal tightening/Whitening ^b^			0.8157
Highly justified	40 (7.22)	31 (6.67)	
Justified	150 (27.08)	117 (25.16)	
Rarely justified	148 (26.71)	123 (26.45)	
Not justified	216 (38.99)	194 (41.72)	
7-Vaginal laser (atrophy)			0.42
Highly justified	45 (8.12)	27 (5.81)	
Justified	139 (25.09)	109 (23.44)	
Rarely justified	139 (25.09)	126 (27.10)	
Not justified	231 (41.70)	203 (43.66)	

^a^ Due to the questionnaire design, “Augmentation of the labia minora” and “G-spot augmentation” were grouped together as a single response item. ^b^ “Laser vaginal tightening” and “Whitening” were also grouped as a single response item. Percentages reflect responses to the combined categories.

**Table 4:** Do you consider undergoing any of the following vulvovaginal esthetic procedures?

Procedure	Single		Married or Have Been Previously Married	
	N	%	N	%
Augmentation of the labia minora/“G-spot” augmentation ^a^	21	3.79	22	4.74
Clitoral surgery	6	1.08	5	1.08
Vaginal rejuvenation	11	1.99	37	7.96
Augmentation of the labia majora	17	3.07	6	1.29
Mons pubis Liposuction	10	1.81	2	0.43
Laser Vaginal tightening	15	2.71	25	5.38
Whitening	84	15.16	45	9.68
Vaginal laser (atrophy)	17	3.07	7	1.51
None	445	80.32	380	81.72

^a^ Combined as a single response item due to questionnaire design.

**Table 5:** Prediction of vaginal microbiota knowledge by vulvovaginal esthetic procedure justification.

Item	Single (*n* = 554)		Married or Have Been Previously Married (*n* = 465)	
	Beta Estimate	95% CI	Beta Estimate	95% CI
Augmentation of the labia minora/“G-spot” augmentation ^a^				
Highly justified	0.2	−0.7836 to 1.103	−1.0	−2.375 to 0.2827
Justified	0.0	−0.6958 to 0.6930	−0.6958 *	−1.369 to −0.02231
Rarely justified	0.2	−0.1798 to 0.5995	−0.2	−0.6304 to 0.2503
Not justified	Ref		Ref	
Clitoral surgery				
Highly justified	0.2	−0.8228 to 1.200	1.0	−0.07449 to 2.129
Justified	0.4	−0.06186 to 0.9045	0.6982 *	0.1151 to 1.281
Rarely justified	0.1	−0.2183 to 0.4836	0.0	−0.3946 to 0.4352
Not justified	Ref		Ref	
Vaginal rejuvenation				
Highly justified	1.054 **	0.3493 to 1.759	0.2	−0.4279 to 0.7999
Justified	0.5809 **	0.1764 to 0.9854	0.2	−0.2865 to 0.6481
Rarely justified	0.5851 **	0.2030 to 0.9673	−0.1	−0.6077 to 0.3153
Not justified	Ref		Ref	
Augmentation of the labia majora				
Highly justified	0.4	−0.6109 to 1.363	1.216 *	0.01080 to 2.422
Justified	−0.1	−0.6711 to 0.5332	0.4	−0.1843 to 1.076
Rarely justified	−0.3	−0.6954 to 0.1666	0.0	−0.4315 to 0.4843
Not justified	Ref		Ref	
Mons pubis liposuction				
Highly justified	0.0	−1.143 to 1.145	−0.7	−1.824 to 0.3294
Justified	0.1	−0.3438 to 0.5521	−0.3	−0.8448 to 0.2890
Rarely justified	−0.1	−0.4829 to 0.2058	−0.3	−0.7158 to 0.1061
Not justified	Ref		Ref	
Laser Vaginal tightening/Whitening ^b^				
Highly justified	−0.2	−0.8797 to 0.4240	−0.1	−0.9142 to 0.6839
Justified	−0.2	−0.6564 to 0.1742	0.0	−0.5007 to 0.4570
Rarely justified	−0.1	−0.4507 to 0.3141	0.4	−0.05376 to 0.8407
Not justified	Ref		Ref	
Vaginal laser (atrophy)				
Highly justified	0.3	−0.2602 to 0.9369	0.1	−0.6907 to 0.8607
Justified	0.1	−0.3055 to 0.5017	−0.1	−0.5726 to 0.4348
Rarely justified	0.3147	−0.07613 to 0.7056	0.143	−0.3005 to 0.5864
Not justified	Ref		Ref	

^a^ “Augmentation of the labia minora” and “G-spot augmentation” were grouped as one item. ^b^ “Laser vaginal tightening” and “Whitening” were grouped as one item; percentages reflect combined responses.* *p* < 0.05; ** *p* ≤ 0.01.

**Table 6:** Prediction of general microbiota knowledge by sociodemographic characteristics.

Sociodemographic Characteristics	Beta Estimate	95% CI
Age		
<20 years	0.2496	−0.1236 to 0.6228
20–30 years	Ref	
30–40 years	0.4217	−0.08347 to 0.9269
40–50 years	0.01902	−0.5264 to 0.5644
>51 years	0.2836	−0.4254 to 0.9925
Marital Status		
Single	Ref	
Married or have been previously married	−0.1069	−0.5861 to 0.3723
Education		
Elementary	−0.08456	−0.9885 to 0.8194
High school	0.08659	−0.2408 to 0.4140
Bachelor	Ref	
Postgraduate	0.09053	−0.3688 to 0.5498
Occupation		
student	Ref	
Employed full-time	0.2882	−0.2044 to 0.7807
Employed part-time	−0.1084	−0.9387 to 0.7219
Self employed	0.389	−0.3629 to 1.141
Unemployed	0.394	−0.09090 to 0.8789
Retired	0.2145	−0.5853 to 1.014
In healthcare		
Yes	1.213 ****	0.8923 to 1.533
No	Ref	
Region		
Central	Ref	
Western	0.155	−0.2672 to 0.5772
Eastern	−0.04831	−0.4731 to 0.3764
Northern	−0.06818	−0.6912 to 0.5549
Southern	−0.159	−0.7020 to 0.3840

**** *p* ≤ 0.0001.

## Data Availability

The data that support the findings of this study are available on request from the corresponding author, E.A.
